# Current Opinion and Practice on Peritoneal Carcinomatosis Management: The North African Perspective

**DOI:** 10.3389/fsurg.2022.798523

**Published:** 2022-03-08

**Authors:** Amine Souadka, Hajar Essangri, Amin Makni, Mourad Abid, Mouna Ayadi, Feriel Ksantini, Zakia Kordjani, Yousri Ballah, Jemila Bouka, Amine Benkabbou, Mohammed Anass Majbar, Basma El Khannoussi, Raouf Mohsine, Saber Boutayeb, Martin Hubner

**Affiliations:** ^1^Surgical Oncology Department, National Institute of Oncology, University Mohammed V, Rabat, Morocco; ^2^Surgical Department A, Rabta Hospital, Tunis, Tunisia; ^3^Surgical Oncology Department, Batna Cancer Institute, Batna, Algeria; ^4^Medical Oncology Department, Salah-Azaiz Institute, Tunis, Tunisia; ^5^Surgical Oncology Department, National Institute of Oncology, Nouakchott, Mauritania; ^6^Pathology Department, National Institute of Oncology, University Mohammed V, Rabat, Morocco; ^7^Medical Oncology Department, National Institute of Oncology, University Mohammed V, Rabat, Morocco; ^8^Department of Visceral Surgery, University Hospital Centre Hospitalier Universitaire Vaudois and University of Lausanne (UNIL), Lausanne, Switzerland

**Keywords:** peritoneal carcinomatosis, LMIC, surveys and questionnaires, North Africa, HIPEC, PIPAC

## Abstract

**Background:**

The status of peritoneal surface malignancy (PSM) management in North Africa is undetermined. The aim of this study was to assess and compare current practice and knowledge regarding PSM and examine satisfaction with available treatment options and need for alternative therapies in North Africa.

**Methods:**

This is a qualitative study involving specialists participating in PSM management in North Africa. The survey analyzed demographic characteristics and current knowledge and opinions regarding PSM management in different institutions. We also looked at goals and priorities, satisfaction with treatment modalities and heated intraperitoneal chemotherapy (HIPEC) usefulness according to specialty, country, years of experience, and activity sector.

**Results:**

One-hundred and three participants responded to the survey (response rate of 57%), including oncologists and surgeons. 59.2% of respondents had more than 10 years experience and 45.6% treated 20–50 PSM cases annually. Participants satisfaction with PSM treatment modalities was mild for gastric cancer (3/10 [IQR 2–3]) and moderate for colorectal (5/10 [IQR 3–5]), ovarian (5/10 [IQR 3–5]), and pseudomyxoma peritonei (5/10 [IQR 3–5]) type of malignancies. Good quality of life and symptom relief were rated as main priorities for treatment and the need for new treatment modalities was rated 9/10 [IQR 8–9]. The perceived usefulness of systemic chemotherapy in first intention was described as high by 42.7 and 39.8% of respondents for PSM of colorectal and gastric origins, while HIPEC was described as highly useful for ovarian (49.5%) and PMP (73.8) malignancies.

**Conclusions:**

The management of PSM in the North African region has distinct differences in knowledge, treatments availability and priorities. Disparities are also noted according to specialty, country, years of expertise, and activity sector. The creation of referral structures and PSM networks could be a step forward to standardized PSM management in the region.

## Introduction

Peritoneal surface malignancy (PSM) is a rare and challenging pathology resulting from the neoplastic progression of different types of primary malignancies. Due to late diagnosis, rapid progression and limited treatment options, PSM was considered until recently as a terminal illness. Cytoreductive surgery (CRS) with hyperthermic intraperitoneal chemotherapy (HIPEC) has emerged as a therapy associated with significant benefits for selected patients with PSM of colorectal (1–3), gastric (4–6), and ovarian origin (7), as well as pseudomyxoma peritonei (8), therefore, changing the historical perception on peritoneal metastases. Other therapies such as perioperative systemic chemotherapy, pressurized intraperitoneal aerosol chemotherapy (PIPAC), and pulsed low dose radiation therapy are also showing promise (9–11), which is resulting in a growing acceptance. However, although a number of centers are venturing in the treatment of peritoneal malignancies and developing specialized programmes, evidence has shown that consideration of this procedure as a treatment option can be low (12). In fact, consideration of these treatment options remains low especially in non-oncologic departments (12), and discrepancies have been noted also between surgical and non-surgical oncologists (13). It appears therefore important to increase awareness and knowledge dissemination regarding these therapies within the cancer care community.

Several studies demonstrated other challenges hindering the acceptance and adoption of PSM treatment strategies, such as the lack of a multidisciplinary approach to patient management (14), the need for specialized training and mentorship but also financial restrictions to establish reference centers for referrals (15–17). These constraints are even worse in low and middle-income countries where cancer care faces additional barriers limiting the access to chemotherapy agents, radiation therapy, and surgical care (18) and where overall healthcare may differ between private and public healthcare systems (19).

North Africa is a diverse region consisting of the five countries forming the Maghreb, namely Morocco, Algeria, Tunisia, Mauritania, and Libya. This region has particular characteristics when it comes to healthcare in general with the public and private sectors playing a complementary role (20) and where cancer incidence has a distincts pattern that completely differs from that of Central and Southern African countries as well as Europe (21). Nonetheless, there is no available data on the knowledge and practices in the management of PSM in this region, except for a few reports (22). The aim of this study was to assess and compare current practice and knowledge regarding PSM and examine satisfaction with available treatment options and need for alternative therapies in North Africa.

## Materials and Methods

### Study Design and Participants

We conducted a qualitative study among a network of specialized North Africa centers which includes all cancer centers involved in the management of PSM in the private and public sector. Representatives from each one of the five countries were contacted in order to help identify institutions undertaking PSM management and a minimum of two participants per center was required. The study was approved by the institutional review boards of all participating centers and was exempt from ethics committee approval as participants gave their consent to participate in the survey. Two similar studies have been previously conducted in a Swiss oncological network (23) and an Indian peritoneal surface malignancy network (24).

The survey included questions on demographics such as country, subspecialty, year of expertise, and number of patients treated personally with PSM. We also examined PSM management in the different institutions, and specialists current knowledge and opinions concerning carcinomatosis. We requested participants to rate on a 5 point likert scale PSM treatment goals and priorities, namely cure, symptom relief, few side effects, few contraindications, inexpensive, or providing a good quality of life. The usefulness of heated intraperitoneal chemotherapy and systemic chemotherapy (in first and second intention) was also assessed, being either poor, moderate or high for PSM of colorectal, ovarian, gastric, and pseudomyxoma peritonei origins. Participants were also required to rate their satisfaction with treatment options on a scale ranging from frustrated: 0 to perfectly happy: 10, as well as the need for new treatment options with 0: no need and 10: urgent need (23, 24). An additional section was included to analyze current practice in the management of PSM originating from pseudomyxoma peritonei tumors. In total, the survey included 29 questions (Appendix 1).

### Statistical Analysis

We reported categorical variables as numbers and percentages, and continuous variables as mean and standard deviations (SD). χ^2^ tests of association were used to compare respondents characteristics and when more than 25% of the subgroups examined were populated by fewer than five respondents, Fisher's exact test or likelihood ratios were performed. Four subgroup analyses were undertaken comparing satisfaction with current treatment modalities, priorities, and perceived need of new treatment options between participants according to specialty (oncologist vs. surgeons), country, years of experience (> or <10 years of experience) and activity sector (public vs. private). Differences were tested with the non-parametric Mann–Whitney *U*-test and Kruskal–Wallis test as adequate. All statistical analyses were performed using SPSS version 25. A significant *P*-value was considered if *p* < 0.05.

## Results

### Participants and Institutions Demographics

The representatives of the four North African countries identified 18 cancer care centers including 180 potentially eligible specialists. In total, 103 participants responded to the survey (response rate of 57%), being either oncologists or surgeons directly involved in the management of PSM in private or public healthcare facilities.

Overall, 59.2% of respondents had more than 10 years experience with 45.6% personally treating 20–50 PC cases annually. There was a significantly higher percentage of surgeons with more than 10 years than oncologists (77.27 vs. 47.5, *P* = 0.045), however, more oncologists reported treating more than 50 PC cases annually (76.9 vs. 43.1 for surgeons, *P* = 0.009). While 88.3 and 71.8% of participating institutions offered systemic chemotherapy and cytoreductive surgery, respectively, only 26.2% had access to HIPEC, with no difference between the private and public sectors (*P* = 0.934). Other treatment modalities such as low dose radiotherapy, pressurized intraperitoneal chemotherapy, were possible in 11.7, 2.9% of institutions, respectively, with a significantly higher percentage of availability in the private sector (*P* = 0.020 and 0.037). Sociodemographic details of the participants in general and according to their subgroups are presented in Table 1.

**Table 1:** Demographics of participating institutions and specialists.

	**Global population**	**Surgeons**	**oncologists**	***P***	**Private**	**Public**	***P***
**Country**							
Morocco	75 (72.8)	32 (72.7)	43 (72.9)	0.154	33 (94.3)	42 (61.8)	**<0.001**
Tunisia	10 (9.7)	7 (15.9)	3 (5.1)		2 (5.7)	8 (11.8)	
Algeria	13 (12.6)	3 (6.8)	10 (16.9)		0	13 (19.1)	
Mauritania	5 (4.9)	2 (4.5)	3 (5.1)		0	5 (7.4)	
**Activity sector**							
Private	35 (25)	21 (47.7)	14 (23.7)	**0.011**	–	–	–
Public	68 (66)	23 (52.3)	45 (76.3)				
**MDT meeting**							
Yes	92 (89.3)	40 (90.9)	52 (88.1)	0.755	28 (80)	64 (94.1)	0.503
No	11 (10.7)	4 (9.1)	7 (11.9)		7 (20)	4 (5.9)	
**Years of experience since board qualification**							
<10 years	41 (39.8)	10 (22.7)	31 (52.5)	**0.045**	8 (22.9)	33 (48.5)	0.248
>10 years	61 (59.2)	34 (77.27)	28 (47.5)		27 (77.1)	35 (55.5)	
**Specialty**							
Oncologist	59 (57.3)	–	–	–	14 (40)	45 (66.2)	**0.011**
Surgeon	44 (42.7)				21 (60)	23 (33.8)	
**Surgical subspecialties**							
General surgeon	29 (72.5)	–	–	–	8 (80)	21 (70)	0.389
Colorectal surgical oncologist	9 (22.5)				1 (10)	8 (26.7)	
Gynecological surgical oncologist	2 (5)				1 (10)	1 (3.3)	
**Patients with PC personally treated annually**							
<20	33 (26)	21 (47.7)	12 (20.3)	**0.009**	13 (37.1)	20 (29.4)	0.723
20–50	47 (45.6)	4 (9.1)	1 (1.7)		15 (42.9)	32 (47.1)	
>50	23 (22.4)	19 (43.1)	46 (76.9)		7 (20)	16 (23.5)	
**Guidelines followed by institution**							
National	12 (11.7)	8 (18.2)	4 (6.8)	0.159	4 (11.4)	8 (11.8)	0.545
French	81 (78.6)	31 (70.5)	50 (84.7)		26 (74.3)	55 (80.9)	
PSOGI	10 (9.7)	5 (11.4)	5 (8.5)		5 (14.3)	5 (7.4)	
**Personal knowledge on PCI calculation method**							
Yes	48 (46.6)	25 (56.8)	23 (39)	0.073	18 (51.4)	30 (44.1)	0.481
No	55 (53.4)	19 (43.2)	36 (61)		17 (48.6)	38 (55.9)	
**Treatment options offered at institution**							
***Cytoreductive surgery***							
Yes	74 (71.8)	32 (72.7)	42 (71.2)	0.863	25 (71.4)	49 (72.1)	0.946
No	29 (28.2)	12 (27.3)	17 (28.8)		10 (28.6)	19 (27.9)	
***HIPEC***							
Yes	27 (26.2)	8 (18.2)	19 (32.2)	0.109	9 (25.7)	18 (26.5)	0.934
No	76 (73.8)	36 (81.8)	40 (67.8)		26 (74.3)	50 (73.5)	
***Systemic chemotherapy***							
Yes	91 (88.3)	32 (72.7)	59 (100)	**<0.001**	29 (82.9)	62 (91.2)	0.330
No	12 (11.7)	12 (27.3)	0 (0)		6 (17.1)	6 (8.8)	
***Intraperitoneal therapy using a catheter***							
Yes	1 (1)	0 (0)	1 (1.7)	1.000	1 (2.9)	0	0.340
No	99 (102)	44 (100)	58 (98.3)		34 (97.1)	68 (100)	
***Pressurized intraperitoneal chemotherapy***							
Yes	3 (2.9)	2 (4.5)	1 (1.7)	0.574	3 (8.6)	0	**0.037**
No	100 (97.1)	42 (95.5)	58 (98.3)		32 (91.4)	68 (100)	
***Low dose radiotherapy***							
Yes	12 (11.7)	5 (11.4)	7 (11.9)	0.938	8 (22.9)	4 (5.9)	**0.020**
No	91 (88.4)	39 (88.6)	52 (88.1)		27 (77.1)	64 (94.1)	
**Management of PC of PMP origin in absence of HIPEC**							
***Chemotherapy***							
Curative	7 (6.8)	0 (0)	7 (11.9)		1 (2.9)	6 (8.9)	
Palliative	4 (3.8)	1 (2.3)	3 (5.1)		0	4 (5.9)	
***Cytoreductive surgery***				**0.003**			0.331
Curative	51 (49.5)	19 (43.2)	32 (54.2)		19 (54.3)	32 (47.1)	
Palliative	4 (3.8)	4 (9.1)	0 (0)		2 (5.7)	2 (2.9)	
Transfert to a specialized center	37 (35.9)	20 (45.5)	17 (28.8)		13 (37.1)	24 (35.3)	

*The bold values mean significant value*.

### Satisfaction

**Figure 1:**
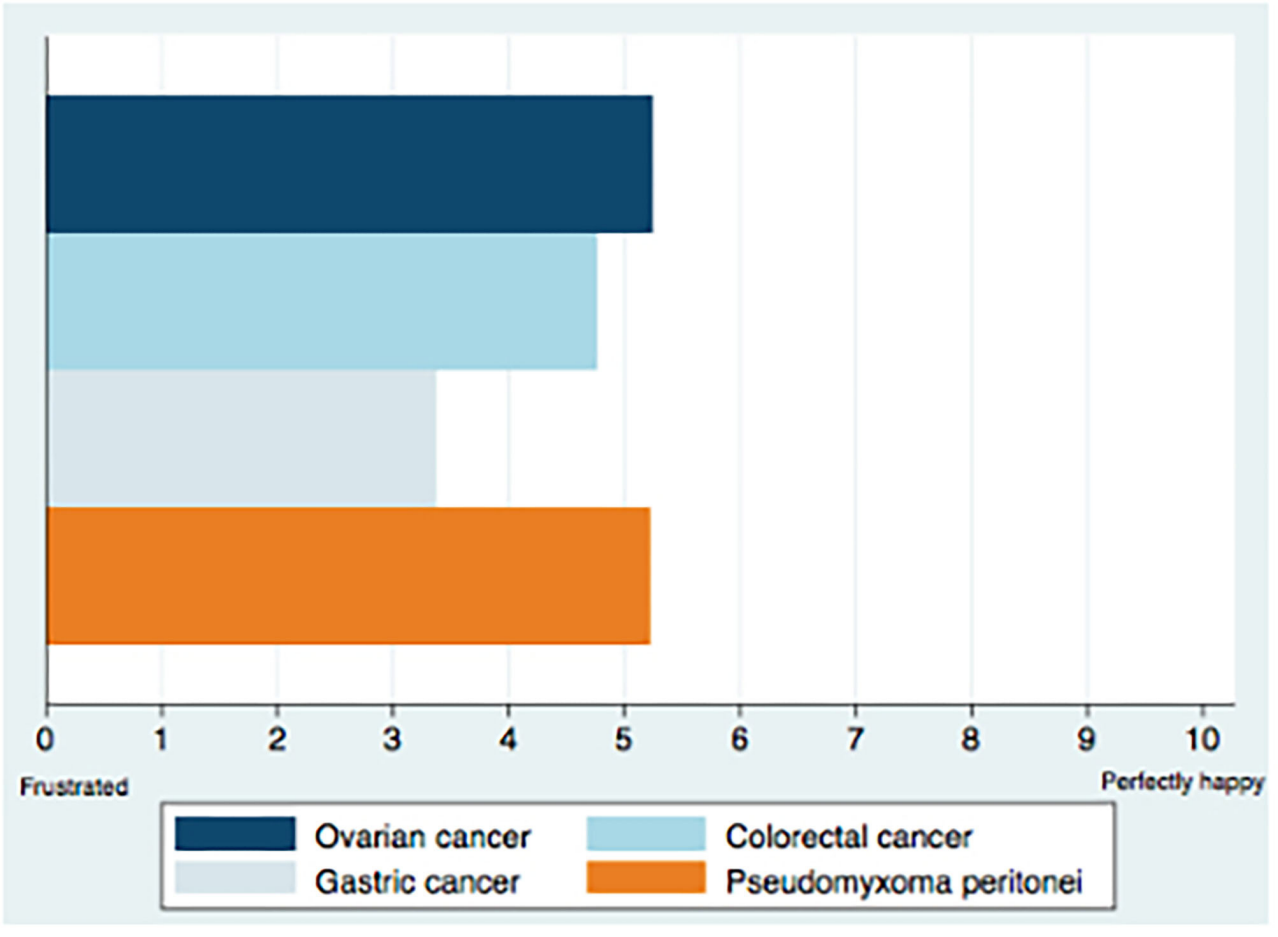
Satisfaction with treatment modalities for PSM.

Details on respondents' satisfaction are described in Figure 1.

Participants satisfaction with PSM treatment modalities was examined according to cancer origin and was mild for gastric cancer (3/10 [IQR 2–3]) and moderate for colorectal (5/10 [IQR 3–5]), ovarian (5/10 [IQR 3–5]), and pseudomyxoma peritonei (5/10 [IQR 3–5]) type of malignancies. The need for new treatment modalities was rated 9/10, [IQR 8–9]. The comparison of the degree of satisfaction according to specialty, country, activity sector, and years of experience showed no statistically significant difference between these subgroups. On the other hand, oncologists reported a higher need for new treatment modalities (9.07 ± 0.98) compared to surgeons (8.02 ± 2.26), *P* = 0.05 (Table 2).

**Table 2:** Subgroup analysis of priorities and degree of satisfaction according to specialty, expertise, country, and activity sector.

	**Specialty**			**Country**					**Activity sector**			**Years of experience**		
	**Surgeon**	**Oncologist**	***P-*** **value**	**Algeria**	**Morocco**	**Mauritania**	**Tunisia**	***P-*** **value**	**Public**	**Private**	***P-*** **value**	** <10 years**	**>10 years**	***P-*** **value**
**Priorities (Mean** **±SD)**														
Cure	3.66 (±1.36)	4.22 (±0.96)	***0.035***	3.77 (±1.23)	4.05 (±1.17)	3.20 (±0.83)	4.10 (±1.28)	*0.205*	3.99 (±1.16)	3.97 (±1.22)	*0.982*	4.20 (±1.05)	3.87 (±1.23)	*0.161*
Symptoms relief	4.55 (±0.79)	4.85 (±0.36)	***0.019***	4.46 (±1.12)	4.75 (±0.49)	4.80 (±0.44)	4.80 (±0.42)	*0.882*	4.69 (±0.65)	4.77 (±0.49)	*0.580*	4.78 (±0.47)	4.67 (±0.67)	*0.434*
Few side effects	3.80 (±1.06)	4.27 (±0.84)	***0.023***	4.08 (±1.03)	4.09 (±0.98)	4.20 (±0.83)	3.80 (±0.91)	*0.775*	4.10 (±1.02)	4 (±0.87)	*0.440*	4.29 (±0.90)	3.90 (±0.99)	*0.045*
Few contraindications	3.55 (±1.02)	4.08 (±0.83)	***0.007***	4.46 (±0.87)	3.76 (±0.95)	3.60 (±0.89)	3.90 (±0.87)	***0.049***	3.93 (±0.95)	3.71 (±0.95)	*0.293*	3.98 (±0.93)	3.75 (±0.96)	*0.252*
Inexpensive	3.39 (±1.14)	3.64 (±1.11)	*0.287*	2.92 (±1.32)	3.67 (±1.08)	4.00 (±1)	3.10 (±0.99)	*0.074*	3.50 (±1.14)	3.60 (±1.11)	*0.694*	3.78 (±1.17)	3.38 (±1.08)	*0.070*
Good quality of life	4.52 (±0.90)	4.86 (±0.34)	***0.031***	4.62 (±1.12)	4.76 (±0,51)	4.20 (±1.30)	4.80 (±0.42)	*0.653*	4.65 (±0.76)	4.86 (±0.35)	*0.224*	4.80 (±0.40)	4.66 (±0.79)	*0.680*
**Satisfaction** **(Mean** **±SD)**														
PC of ovarian origin	5.61 (±2.83)	5 (±2.66)	*0.301*	5.23 (±2.97)	5.33 (±2.75)	5 (±2.55)	4.90 (±2.80)	*0.963*	5.25 (±2.79)	5.29 (±2.66)	*0.958*	5.05 (±2.91)	5.39 (±2.65)	*0.498*
PC of colorectal origin	5.05 (±2.77)	4.58 (±2.50)	*0.371*	5.23 (±3.29)	4.61(±2.48)	3.60 (±3.28)	6 (±2.26)	*0.274*	4.81 (±2.66)	4.71 (±2.58)	*0.836*	4.34 (±2.75)	5.05 (±2.53)	*0.126*
PC of gastric origin	3.30 (±2.37)	3.46 (±2.71)	*0.984*	3.46 (±3.86)	3.40 (±2.33)	2.40 (±1.81)	3.70 (±2.79)	*0.722*	3.56 (±2.73)	3.06 (±2.19)	*0.486*	3.54 (±2.81)	3.31 (±2.42)	*0.793*
PC of PMP origin	5.68 (±3.35)	4.90 (± 2.82)	*0.213*	6 (±2.94)	5.08 (±3.06)	3 (±2.64)	6.50 (±3.06)	*0.138*	4.93 (±3.09)	5.83 (±2.98)	*0.157*	4.93 (±3.00)	5.49 (±2.65)	*0.396*
**The need of new treatment modalities**	8.02 (±2.26)	9.07 (±0.98)	***0.05***	8.92 (±1.93)	8.64 (±1.74)	8.60 (±1.14)	8.20 (±1.61)	*0.434*	8.70 (±1.50)	8.49 (±2.07)	*0.930*	8.71 (±1.66)	8.57 (±1.76)	*0.503*

*The bold values mean significant value*.

### Goals and Priorities

**Figure 2:**
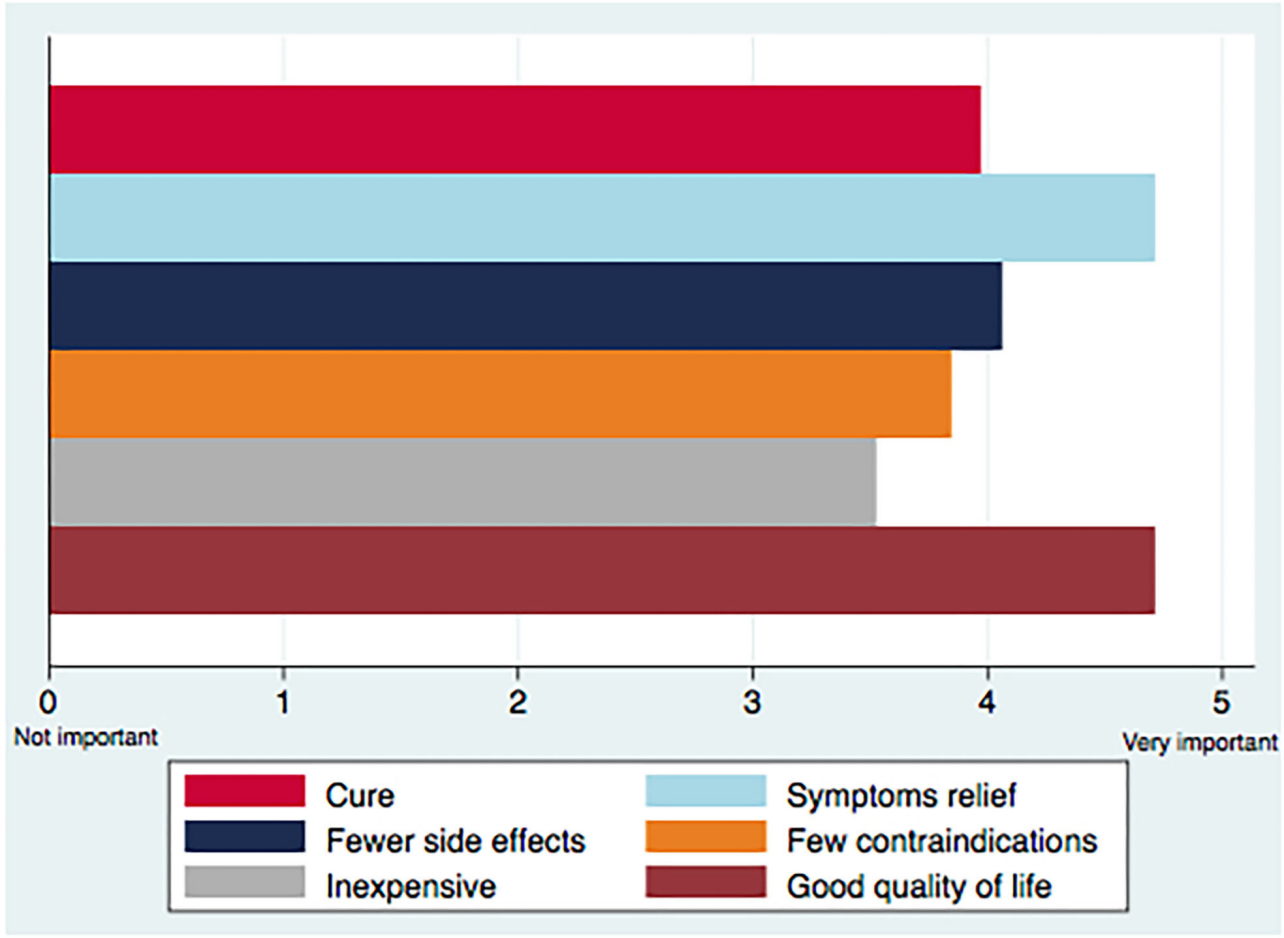
Main priorities in the treatment of peritoneal carcinomatosis.

79.6 and 76.7% of participants rated good quality of life and symptom relief as very important, while few contraindications and inexpensive treatments were rated less important (Figure 2).

Looking at treatment goals according to the different subgroups, oncologists rated a statistically significant higher degree of priority for all elements, except for inexpensiveness (*P* = 0.287), while prioritizing fewer contraindications was different between countries (*P* = 0.049). No significant variation was established in the priorities according to years of experience and activity sector (Table 2).

### Usefulness of Systemic Chemotherapy and HIPEC in Resectable Peritoneal Metastasis

Overall, the perceived usefulness of systemic chemotherapy in first intention was described as high by 42.7 and 39.8% of respondents for PSM of colorectal and gastric origins, while HIPEC was described as highly useful for ovarian (49.5%) and PMP (73.8) malignancies.

Subgroup analysis yielded a significant difference between surgeons and oncologists, with 91.5% oncologists rating systemic chemotherapy as a second line intention for PC of ovarian origin as highly useful, compared to 75% of surgeons (*P* = 0.044). Differences were also noted in the perceived usefulness for systemic chemotherapy in first line intention for PC of colorectal (*P* = 0.030) and gastric origin (*P* = 0.029) between specialists from the private and public sector.

Details on the usefulness of chemotherapy and HIPEC in the management of resectable peritoneal metastasis are demonstrated in Table 3.

**Table 3:** Perceived clinical usefulness of systemic chemotherapy and HIPEC in resectable peritoneal metastasis.

	**Global population**			**Surgeons**			**Oncologists**			***P*** **-value**	**Public**			**Private**			***P-*** **value**
	**Poor**	**Moderate**	**High**	**Poor**	**Moderate**	**High**	**Poor**	**Moderate**	**High**		**Poor**	**Moderate**	**High**	**Poor**	**Moderate**	**High**	
**Systemic chemotherapy**																	
***Colorectal origin***																	
First-line intention	14 (13.6)	45 (43.7)	44 (42.7)	9 (20.5)	21 (47.7)	14 (31.8)	5 (8.5)	24 (40.7)	30 (50.8)	*0.079*	8 (11.8)	36 (52.9)	24 (35.3)	6 (17.1)	9 (25.7)	20 (57.1)	***0.030***
Second-line intention	12 (11.7)	37 (35.9)	54 (52.4)	6 (13.6)	13 (29.5)	25 (56.8)	6 (10.2)	24 (40.7)	29 (49.2)	*0.494*	8 (11.8)	23 (33.8)	37 (54.4)	4 (11.4)	14 (40)	17 (48.6)	*0.820*
***Gastric origin***																	
First-line intention	32 (31.1)	30 (29.1)	41 (39.8)	15 (34.1)	11 (25)	18 (40.9)	17 (28.8)	19 (32.2)	23 (39)	*0.705*	24 (35.3)	14 (20.6)	30 (44.1)	8 (22.9)	16 (45.7)	11 (31.4)	***0.029***
Second-line intention	41 (40.8)	31 (30.1)	30 (29.1)	17 (38.6)	12 (27.3)	15 (34.1)	25 (42.4)	19 (32.2)	15 (25.4)	*0.625*	28 (41.2)	19 (27.9)	21 (30.9)	14 (40)	12 (34.3)	9 (25.7)	*0.768*
***Ovarian origin***																	
Second-line intention	6 (5.8)	10 (9.7)	87 (84.5)	5 (11.4)	6 (13.6)	33 (75)	1 (1.7)	4 (6.8)	54 (91.5)	***0.044***	3 (4.4)	8 (11.8)	57 (83.8)	3 (8.6)	2 (5.7)	30 (85.7)	*0.447*
Third-line intention	40 (39.2)	32 (31.3)	30 (29.4)	16 (36.6)	12 (27.3)	16 (36.6)	24 (41.3)	20 (34.4)	14 (24.3)	*0.6*	28 (41.2)	19 (27.9)	21 (30.9)	12 (35.2)	13 (38.2)	9 (26.4)	*0.72*
**HIPEC**																	
Ovarian origin	11 (10.7)	41 (39.8)	51 (49.5)	5 (11.4)	13 (29.5)	26 (59.1)	6 (10.2)	28 (47.5)	25 (42.4)	*0.175*	7 (10.3)	26 (38.2)	35 (51.5)	4 (11.4)	15 (42.9)	16 (45.7)	*0.858*
Colorectal origin	22 (21.4)	38 (36.9)	43 (41.7)	9 (20.5)	17 (38.6)	18 (40.9)	13 (22)	21 (35.6)	25 (42.4)	*0.949*	15 (22.1)	24 (35.3)	29 (42.6)	7 (20)	14 (40)	14 (40)	*0.894*
Gastric origin	47 (45.6)	40 (38.8)	16 (15.5)	18 (40.9)	17 (38.6)	9 (20.5)	29 (49.2)	23 (39)	7 (11.9)	*0.455*	30 (44.1)	28 (41.2)	10 (14.7)	17 (48.6)	12 (34.3)	6 (17.1)	*0.790*
PMP origin	7 (6.8)	20 (19.4)	76 (73.8)	4 (9.1)	9 (20.5)	31 (70.5)	3 (5.1)	11 (18.6)	45 (76.3)	*0.689*	4 (5.9)	11 (16.2)	53 (77.9)	3 (8.6)	9 (25.7)	23 (65.7)	*0.416*

*The bold values mean significant value*.

## Discussion

PSM can occur as a primary malignancy, or originate from the progression of an array of tumors, such as gastric, colorectal, or ovarian cancers. In the case of metastatic spreading, this can be justified using either one of the two main hypotheses whereby tumor progression can be promoted by anatomical factors such as the local spreading of tumor cells after breaching the initial site or by the “seed-and-soil” hypothesis implying the specific tropism of tumor cells circulating in the lymphatic or vascular systems to certain organs. However, unknown alternative biological routes may also exist (27–29). As such, management options also vary according to tumor origins, and whether the aim of treatment is curative or palliative. This study depicts the heterogeneous knowledge on the management of PSM, differences in priorities, and lack of guideline use and standardization. Furthermore, it underlines potential differences between specialists according to specialty, country, years of expertise, and activity sector. Efforts are needed to disseminate knowledge and awareness through the creation of networks and referral structures in order to offer optimal care to the largest possible number of patients.

This survey targeted the network of cancer care centers involved in the management of PSM in North Africa, which comprises oncologists and surgeons from Morocco, Algeria, Tunisia, and Mauritania. Overall, almost half of participants reported personally treating 20–50 patients annually. However, access to HIPEC or other therapies such as PIPAC or low dose radiotherapy was poor. In case of HIPEC unavailability, 35.9% of respondents choose patient transfer to a specialized center as an alternative, while only 7.6% of respondents sought either palliative chemotherapy or cytoreductive surgery as a substitute, which could convey an overall good understanding of PSM' changing prognosis and the acceptance of HIPEC as a potentially curative treatment option.

The results from our study were comparable to the two previous surveys conducted in the Swiss (23) and Indian networks (24). In fact, similarities were noted between the three networks, with a particular lack of satisfaction for gastric cancer compared to other origins of malignancies, which could be due to the extremely bad prognosis of PSM of this origin (30). That being said, the need for new treatment modalities was also rated as high. On the other hand, although the cost of treatment was the least important treatment goal in all three surveys, other priorities differed with a higher importance given to good quality of life by North African specialists, while cure was ranked as the main goal in the other studies. This could uncover a common misconception about quality of life decline in the treatment of PSM (31). As regards the degree of usefulness of chemotherapy and HIPEC, all three networks recognize a moderate to high usefulness of these therapies in ovarian and colorectal cancer as a first or second line treatment, with less perceived advantages for PC of gastric origin despite regional recommendations that conform to the international guidance (32). We also examined the degree of satisfaction and perceived usefulness of chemotherapy and HIPEC in the case of primary PC originating from pseudomyxoma peritonei, which illustrated a moderate degree of satisfaction and high perceived degree of effectiveness for HIPEC.

Previous studies on the acceptance of PC therapies portrayed a substantial variation in opinions according to factors such as specialty, experience, implementation of multidisciplinary tumor boards and availability of expert centers (13, 26, 33, 34), which could contribute to the underutilization of these treatments. Accordingly, we compared potential differences between specialists and according to years of expertise, activity sector as well as country. When comparing surgeons and oncologists' point of views, some differences in priorities and need for new therapies have been observed. However, no disagreement has been noted regarding chemotherapy and HIPEC use, except for PC of ovarian origin, for which HIPEC indications are still a subject of controversy (25, 35). The ongoing debate between oncologists, surgeons, and other non-oncologic specialists regarding PC has been established in previous studies (12, 13), with suggestions that a multidisciplinary discussion among different specialties is of utmost importance to overcome personal preferences and knowledge gaps, while deciding for treatment or even referral suitability (36, 37). In our study, 89.3% of specialists reported discussing their cases in multidisciplinary team meetings.

In resource restricted contexts such as in the North African region, the prioritization and indications for the curative treatment may also face the constraints of limited critical care and surgical resources management and expertise (22, 38–41). In fact, while only 26.2% of our respondents reported having access to HIPEC, a similar study on practice patterns in PC from the USA identified 65.8% of its participants as having easy access to a HIPEC and CRS specialists, with 42.7% being in the same hospital (33). In such settings, the world health organization (WHO) has identified the private sector as an additional asset allowing the provision of infrastructure, support services, medicines and medical products, as well as a solution to some financial challenges (42). As such, we choose to include the private sector in our analysis, which indeed demonstrated that the significantly higher availability in the private sector of some therapies such as pressurized intraperitoneal chemotherapy and low dose radiotherapy didn't necessarily yield a higher level of satisfaction in the available treatment modalities. Regardless of the activity sector, the level of expertise is considered a better reflection (22), especially as the PSM treatment learning curve can be steep and complex (43).

PSM management has undergone a tremendous change and is still subject to evolving with ongoing randomized clinical trials and emerging new techniques. However, this progress is also perceived as chaotic with many methodological variations according to patients, type and stage of carcinomatosis (44). This is why there is a need for strong collaborations between different specialists and even countries which were more successful in some areas, in order to ensure knowledge dissemination. The creation of organizations could also be a step forward toward consolidating efforts for the effective implementation of these innovative therapies as per the IDEAL framework, as well as developing guidelines regarding patient selection and therapy standardization (44–46). In our context, only 9.7% of respondents reported complying with the peritoneal surface oncology group international guidelines, which could reflect the need for a regional dialogue and a possible adaptation of these recommendations to the North African context, especially as no dissimilarities have been noted between four countries.

This study is exploratory research with some limitations. Firstly, participants' selection was done through designated referees from each country who were in charge of contacting centers, which could result in some selection and reach bias. That being said, all declared cancer care centers in the region were contacted. Also, gynecological surgeons were under-represented in this group, which is due to the fact that gynecological oncological surgery is mostly performed by general oncological surgeons in the North African setting.

Notwithstanding these limitations, this is the first study addressing the current opinions in the North African region, while capturing the differences between specialties and activity sectors, as well as reflecting the real picture of practice in a region where little published data is available on the management of PC.

## Conclusion

The assessment of current opinions and knowledge on peritoneal surface malignancy in the North African region indicates a low satisfaction with currently available treatment options and different perspectives between medical oncologists and surgeons. In spite of that, there was a notable homogeneity in treatment practices, which could reflect the possibility of developing a North African collaboration with regional guidelines that allow the adaptation of international recommendations to peritoneal surface malignancy management and that could serve as a template for other low and middle income settings.

## Data Availability Statement

The original contributions presented in the study are included in the article/Supplementary Material, further inquiries can be directed to the corresponding author/s.

## Author Contributions

AS, SB, and MH have contributed to the conception and the design of the study. AS, SB, HE, AM, MAb, and JB have contributed to the acquisition of the data. AS, SB, and HE contributed to the analysis and the interpretation of data. AS and HE wrote the first draft. MM, AB, and MH critically reviewed the draft for important intellectual content. MAy, FK, ZK, YB, BE, and RM were involved in revising critically the corrected manuscript. All authors read and gave the final approval of the version to be published.

## Funding

Publication fees supported by the Association Cancer Espoir, Rabat, Morocco.

## Conflict of Interest

The authors declare that the research was conducted in the absence of any commercial or financial relationships that could be construed as a potential conflict of interest.

## Publisher's Note

All claims expressed in this article are solely those of the authors and do not necessarily represent those of their affiliated organizations, or those of the publisher, the editors and the reviewers. Any product that may be evaluated in this article, or claim that may be made by its manufacturer, is not guaranteed or endorsed by the publisher.
