# Supplementary material for: Current Opinion and Practice on Peritoneal Carcinomatosis Management: The North African Perspective
Source: Front Surg. 2022 Mar 8;9:798523. doi: 10.3389/fsurg.2022.798523 (PMC8957835; doi:10.3389/fsurg.2022.798523)
Supplement: Supplementary file 1 [file Data_Sheet_1.PDF]

Appendix 1:

Survey : Current opinion and practice on peritoneal surface malignancy (PSM) management: the north African perspective

1. What is your medical specialty ?

    Oncological surgeon

    Gynecological surgeon

    Colorectal surgeon

    General surgeon

    Oncologist

2. For how long have you been working since board qualification ?

    < 10 years

    > 10 years

3. In what sector do you work ?

    Private

    Public

4. In what country do you work ?

    Algeria

Morocco

Tunisia

Mauritania

Libya

5. What’s the number of patients that you’re involved in the care of annually and that have PSM ?

   < 20

   20 - 50

   > 50

6. Are these treatment options available in your institution for PSM patients (several answers are possible)

   Systemic chemotherapy

   Cytoreductive surgery

   Heated intraperitoneal chemotherapy

   Low dose radiotherapy

   Intraperitoneal therapy using a catheter

   Pressurized intraperitoneal chemotherapy

   None

7. What's the annual number of hyperthermic intraperitoneal chemotherapy (HIPEC) procedures at your institution?

   0

   < 10

   10 - 20

   20-50

   > 50

8. Are you familiar with Sugarbaker´s Peritoneal Carcinomatosis Index (PCI) ?

   Yes

   No

9. What are the minimal standard investigations you would order for PSM regardless of its origin ? (several answers are possible)

   Thoracoabdominopelvic CT

   Digestive endoscopy

   Immunohistochemistry

   Tumor markers

   PET Scan

   MRI

The next few questions concern the management of PSM of pseudomyxoma peritonei origin. Please feel free to answer all questions regardless of your medical speciality.

10. What recommendations do you use for treating peritoneal pseudomyxoma in your routine practice?

    National

    French

    PSOGI

11. What are the minimal standard investigations you would order for PSM of pseudomyxoma peritonei origin ? (several answers are possible)

    Thoracoabdominopelvic CT

    Digestive endoscopy

    Immunohistochemistry

    Tumor markers

    PET Scan

    MRI

12. In case of HIPEC unavailability, and faced with resectable PSM, what would be your management for a PSM of peritoneal pseudomyxoma?

    Curative chemotherapy

    Palliative chemotherapy

    Curative cytoreductive surgery

Palliative cytoreductive surgery

Transfert to a specialized center

The next questions concern the systemic treatment of PSM of various origins. Please feel free to answer all questions regardless of your medical speciality.

13. What are your main goals/priorities for the treatment of your patients with PSM?

Please rate the importance of following statements on a Likert scale from 0 (not important) to 5 (very important).

Cure

Symptom relief

Few side effects

Few contraindications

Inexpensive

Good quality of life

14. How would you describe the clinical usefulness of systemic chemotherapy in FIRST LINE intention treatment of resectable PSM of COLORECTAL origin?

As first line treatment

Poor

Moderate

High

15. How would you describe the clinical usefulness of systemic chemotherapy in SECOND LINE intention treatment of resectable PSM of COLORECTAL origin?

As second line treatment

Poor

Moderate

High

16. How would you describe the clinical usefulness of systemic chemotherapy in FIRST LINE intention treatment of resectable PSM of GASTRIC origin?

As first line treatment

Poor

Moderate

High

17. How would you describe the clinical usefulness of systemic chemotherapy in SECOND LINE intention treatment of resectable PSM of GASTRIC origin?

As second line treatment

Poor

Moderate

High

18. How would you describe the clinical usefulness of systemic chemotherapy in SECOND LINE intention treatment of resectable PSM of OVARIAN origin?

As second line treatment

Poor

Moderate

High

19. How would you describe the clinical usefulness of systemic chemotherapy in THIRD LINE intention treatment of resectable PSM of OVARIAN origin?

As third line treatment

Poor

Moderate

High

The next few questions concern the surgical treatment of PSM with Cytoreductive Surgery and Hyperthermic Intraperitoneal Chemotherapy (HIPEC). Please feel free to answer all questions regardless of your medical speciality.

20. How would you describe the clinical utility of hyperthermic intraperitoneal chemotherapy (CHIP) in the treatment of isolated resectable PSM of OVARIAN origin?

Poor

Moderate

High

21. How would you describe the clinical utility of hyperthermic intraperitoneal chemotherapy (CHIP) in the treatment of isolated resectable PSM of COLORECTAL origin?

Poor

Moderate

High

22. How would you describe the clinical utility of hyperthermic intraperitoneal chemotherapy (CHIP) in the treatment of isolated resectable PSM of GASTRIC origin?

Poor

Moderate

High

23. How would you describe the clinical utility of hyperthermic intraperitoneal chemotherapy (CHIP) in the treatment of isolated resectable PSM of PSEUDOMYXOMA PERITONEI origin?

Poor

Moderate

High

The next questions try to evaluate your satisfaction with available treatment options for PSM. Please feel free to answer all questions regardless of your medical speciality.

24. Please indicate on a Visual Analogue Scale the need (0= no need, 10= urgent need) for new treatment options for PSM:

0	1	2	3	4	5	6	7	8	9	10
○	○	○	○	○	○	○	○	○	○	○

25. Please indicate on a Visual Analogue Scale from 0 (=frustrated) to 10 (=perfectly happy) your satisfaction with available treatment options for PSM of OVARIAN origin:

0	1	2	3	4	5	6	7	8	9	10
○	○	○	○	○	○	○	○	○	○	○

26. Please indicate on a Visual Analogue Scale from 0 (=frustrated) to 10 (=perfectly happy) your satisfaction with available treatment options for PSM of COLORECTAL origin:

0	1	2	3	4	5	6	7	8	9	10
○	○	○	○	○	○	○	○	○	○	○

27. Please indicate on a Visual Analogue Scale from 0 (=frustrated) to 10 (=perfectly happy) your satisfaction with available treatment options for PSM of GASTRIC origin:

0	1	2	3	4	5	6	7	8	9	10
○	○	○	○	○	○	○	○	○	○	○

28. Please indicate on a Visual Analogue Scale from 0 (=frustrated) to 10 (=perfectly happy) your satisfaction with available treatment options for PSM of PSEUDOMYXOMA PERITONEI origin:

0	1	2	3	4	5	6	7	8	9	10
○	○	○	○	○	○	○	○	○	○	○

29. Do you have access to a multidisciplinary team (MDT) meeting dedicated to discussing your digestive and / or gynecomammary oncology patients?

No

Digestive MDT meeting only

Gynecomammary MDT meeting only

Digestive and gynecomammary MDT meeting
