# Supplementary material for: Current Opinion and Practice on Peritoneal Carcinomatosis Management: The North African Perspective
Source: Front Surg. 2022 Mar 8;9:798523. doi: 10.3389/fsurg.2022.798523 (PMC8957835; doi:10.3389/fsurg.2022.798523)
Supplement: Supplementary file 2 [file Data_Sheet_2.PDF]

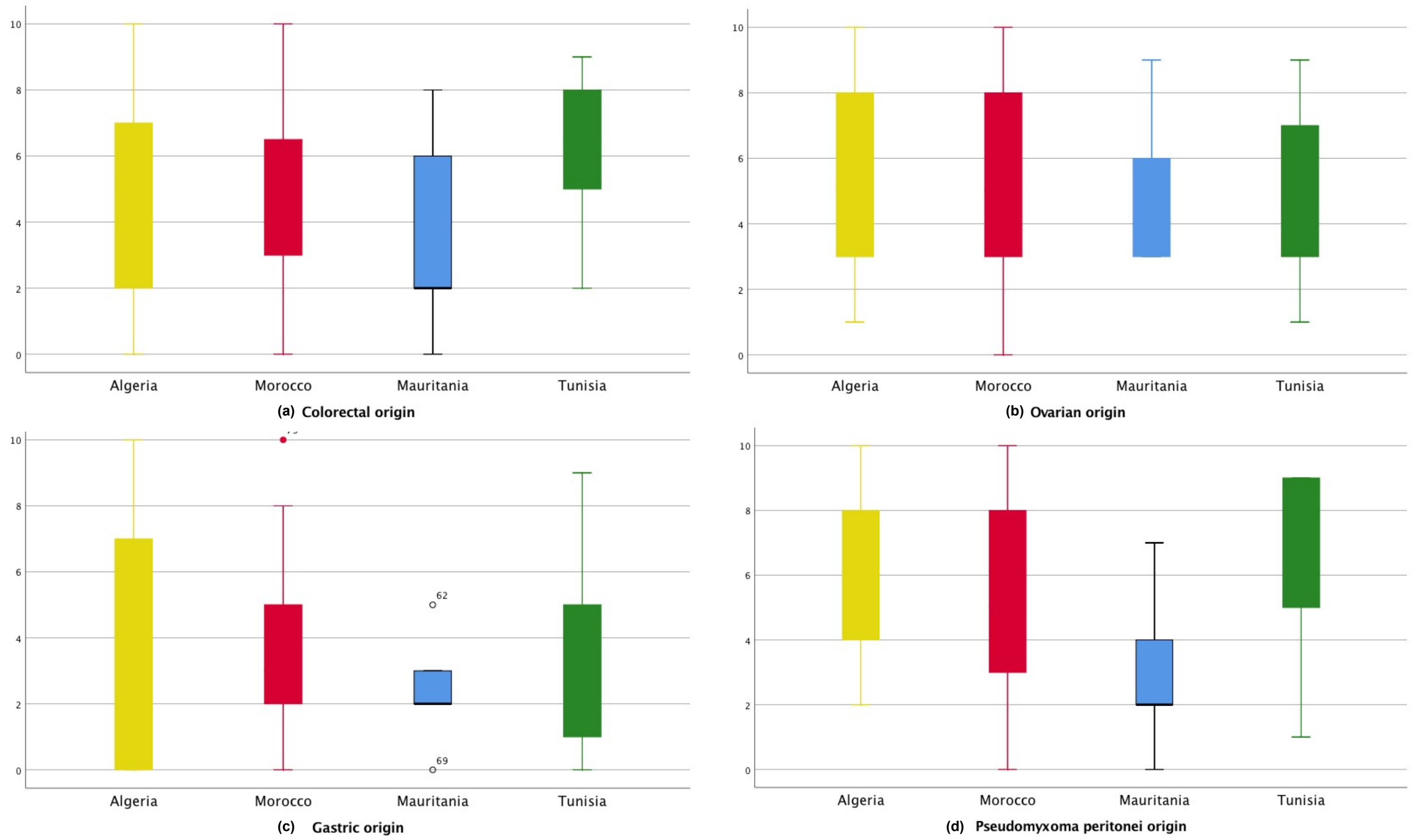

Figure 1 : Satisfaction with available treatment options according to country for peritoneal carcinomatosis of different origins

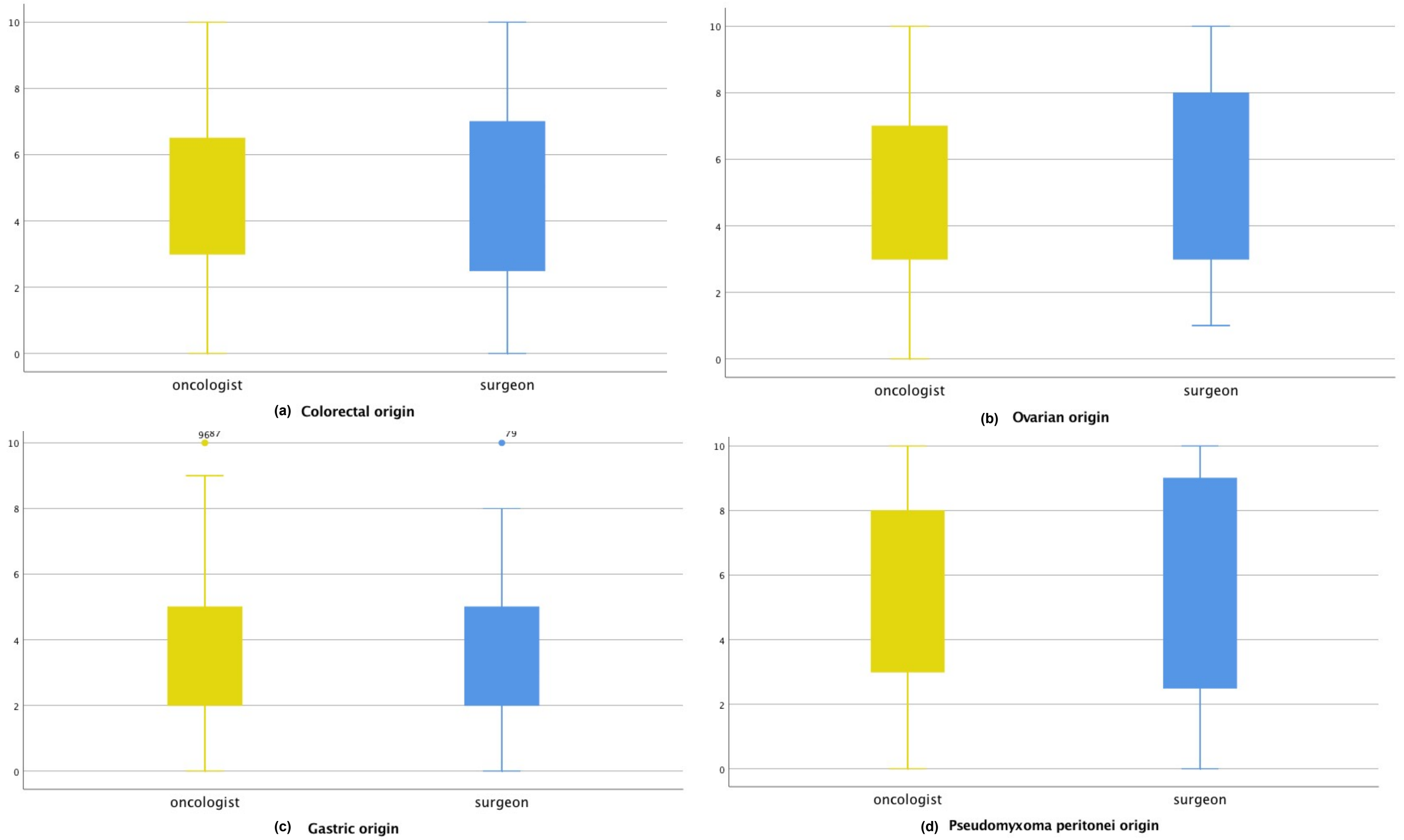

Figure 2 : Satisfaction with available treatment options according to specialty for peritoneal carcinomatosis of different origins

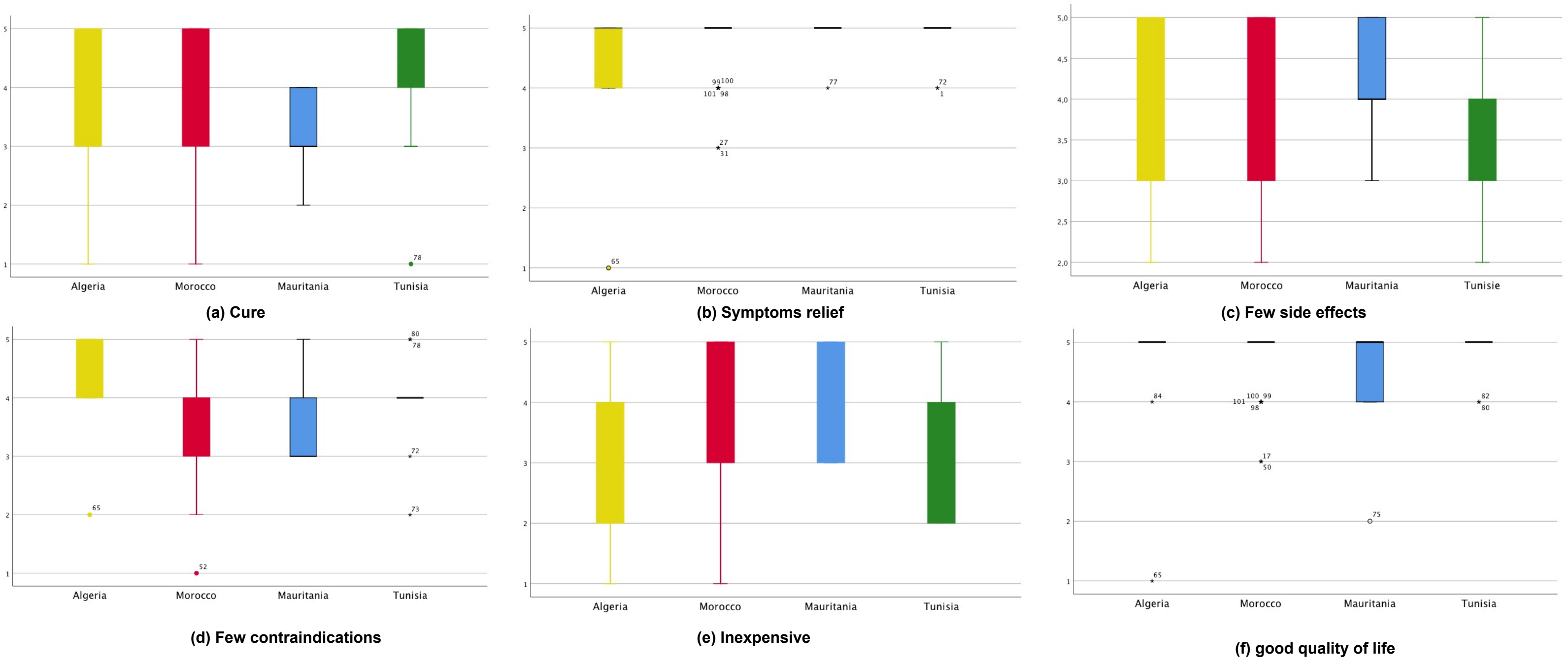

(a) Cure
(b) Symptoms relief
(c) Few side effects
(d) Few contraindications
(e) Inexpensive
(f) good quality of life

Figure 3 : Main goals for the treatment of patients with peritoneal carcinomatosis according to country

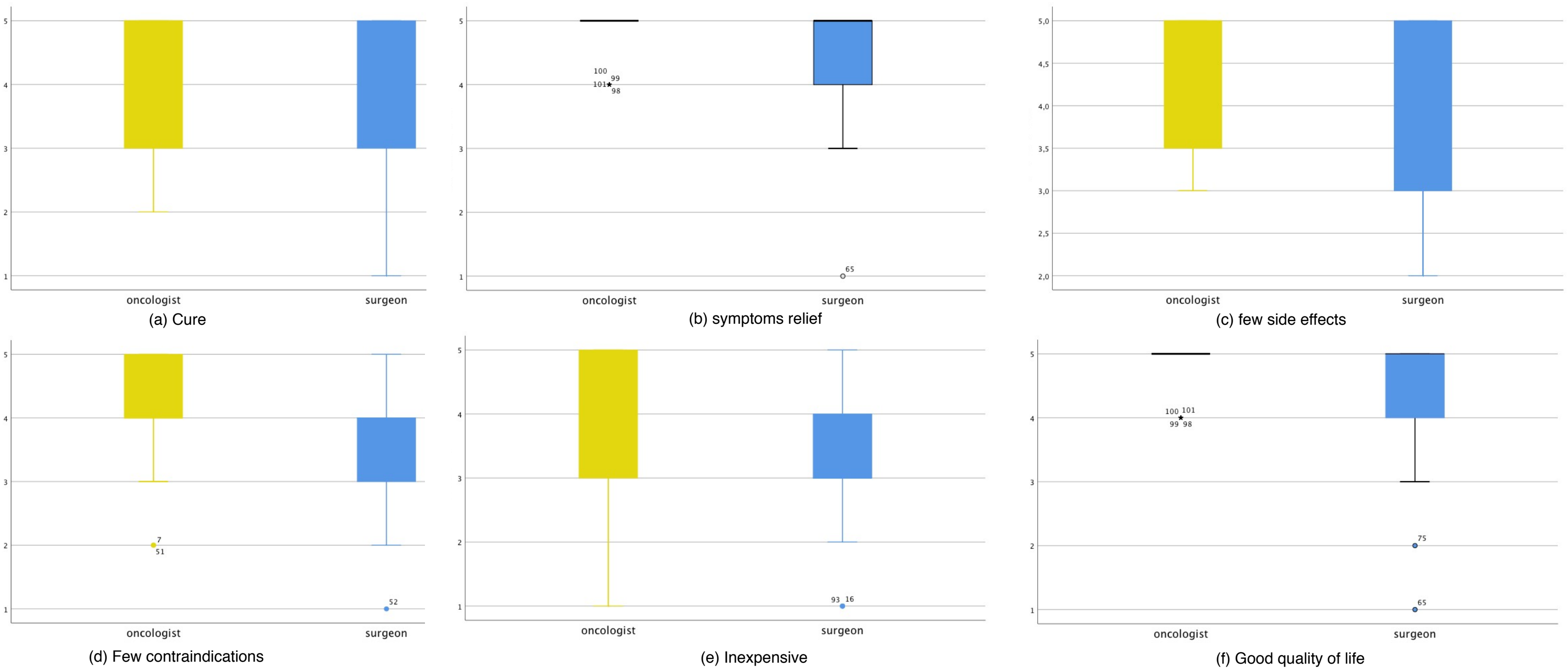

(a) Cure
(b) symptoms relief
(c) few side effects
(d) Few contraindications
(e) Inexpensive
(f) Good quality of life

Figure 4 : Main goals for the treatment of patients with peritoneal carcinomatosis according to specialty
